# Regional Anesthesia and the Perioperative Inflammatory Window in Cancer Surgery: From Surgical Stress to Immunometabolic Reprogramming

**DOI:** 10.3390/cancers18071158

**Published:** 2026-04-03

**Authors:** Tomasz Reysner, Malgorzata Reysner

**Affiliations:** 1 Pathophysiology of Pain Unit, Department of Anesthesiology and Intensive Therapy, Poznan University of Medical Sciences, ul. Przybyszewskiego 49, 60-355 Poznań, Poland; treysner@ump.edu.pl; 2 Department of Clinical Anesthesiology and Pain Management, Poznan University of Medical Sciences, ul. 28 Czerwca 1956 135/147, 61-701 Poznań, Poland

**Keywords:** regional anesthesia, cancer surgery, perioperative inflammation, surgical stress response, immunometabolism, tumor microenvironment, anesthetic technique, perioperative oncology

## Abstract

The impact of anesthetic technique on long-term oncologic outcomes remains controversial. While early observational data suggested that regional anesthesia might reduce cancer recurrence, large randomized trials have failed to demonstrate consistent survival benefits. This apparent contradiction may not reflect biological neutrality, but rather a mismatch between trial design and the inflammatory biology of the perioperative period. Surgical resection provokes an acute and intense inflammatory surge characterized by sympathetic activation, cytokine release, neutrophil extracellular trap formation, endothelial activation, and transient suppression of cellular immunity. During this perioperative inflammatory window, circulating tumor cells encounter a biologically permissive microenvironment that may facilitate immune evasion, adhesion, and early metastatic niche establishment. The magnitude of this inflammatory response varies across patients and may represent a critical, yet under-recognized, determinant of tumor–host dynamics. Anesthetic and analgesic strategies influence this inflammatory cascade. By attenuating nociceptive signaling and sympathetic activation, regional anesthesia may modulate perioperative immune and immunometabolic pathways. However, it should not be framed as an anti-cancer therapy per se, but rather as a potential regulator of the transient inflammatory milieu that shapes early oncologic biology. We propose that prior neutral trials may reflect methodological misalignment, including heterogeneous tumor populations, absence of inflammatory stratification, and reliance on distant survival endpoints without mechanistic correlates. Future investigations should integrate perioperative immune phenotyping, inflammatory biomarkers, and tumor subtype stratification to determine whether modulation of acute surgical inflammation meaningfully alters early tumor–host interactions. Reconceptualizing the perioperative period as a biologically active inflammatory interface may refine the anesthesiologist’s role within perioperative oncology and open new avenues for precision-based perioperative modulation.

## Simple Summary

Cancer surgery can unintentionally create conditions that help cancer cells survive and spread. The body reacts to surgery with a strong stress and inflammatory response, which can temporarily weaken the immune system. During this short period, circulating cancer cells may more easily attach to tissues and form new tumors. This review explores whether the type of anesthesia used during surgery can influence this process. In particular, regional anesthesia may reduce the body’s stress response and inflammation, helping to maintain a more balanced immune environment. However, current clinical studies have not consistently shown improved long-term cancer outcomes. We suggest that this may be because previous research did not focus on the immediate biological changes after surgery. Future studies should better evaluate how anesthesia affects inflammation and immune function in the perioperative period to understand its true role in cancer care.

## 1. Introduction

The hypothesis that anesthetic technique may influence long-term oncologic outcomes has generated sustained interest over the past two decades. Early retrospective analyses suggested that regional anesthesia and analgesia could be associated with reduced cancer recurrence and improved survival following oncologic surgery. These findings, biologically plausible and clinically provocative, catalyzed a wave of mechanistic investigations and randomized trials [1]. Yet large prospective studies have failed to demonstrate consistent survival advantages, leading to skepticism and, in some circles, to the dismissal of the concept altogether [2].

This apparent discrepancy between biological plausibility and clinical evidence may reflect a deeper conceptual misalignment. Cancer recurrence and long-term survival are distant, heterogeneous, and multifactorial endpoints, shaped by tumor biology, adjuvant therapies, genetic factors, and host immunity over years. In contrast, anesthetic interventions act within a narrow and temporally confined perioperative window [3]. It is during this period—characterized by surgical stress, sympathetic activation, inflammatory surge, and transient immune suppression—that circulating tumor cells may either be eliminated or gain a foothold for micrometastatic progression. Framing the role of regional anesthesia solely in terms of five-year recurrence may therefore obscure its more immediate and biologically relevant effects [4]. Importantly, this perioperative inflammatory surge represents a transient but biologically potent driver of tumor–host interactions, positioning acute inflammation as a central mechanistic interface between surgery and cancer progression.

Regional anesthesia may therefore be best conceptualized not as an anti-cancer therapy [5], but as a modulator of the perioperative immunometabolic milieu [6].

### 1.1. Methods

This narrative review was conducted to synthesize current evidence on the potential influence of anesthetic techniques on perioperative tumor biology and long-term oncologic outcomes, with a particular focus on the perioperative immunometabolic window.

A structured literature search was performed in the PubMed, Scopus, and Web of Science databases for studies published between January 2000 and February 2026. The following key terms and combinations were used: “regional anesthesia”, “general anesthesia”, “cancer recurrence”, “oncologic outcomes”, “perioperative immune response”, “immunosuppression”, “neutrophil extracellular traps”, “immunometabolism”, “inflammation”, and “surgical stress response”.

The initial search yielded approximately 1123 records. After removal of duplicates, titles and abstracts of approximately 862 articles were screened for relevance. Studies were considered eligible if they addressed the relationship between anesthetic techniques, perioperative inflammation, immune modulation, or oncologic outcomes.

Eligible publications included randomized controlled trials, prospective and retrospective clinical studies, systematic reviews, meta-analyses, and relevant translational or mechanistic studies. Articles not available in English and case reports were excluded.

Full-text assessment was performed for approximately 210 articles, of which around 120 studies were considered most relevant and included in the narrative synthesis. Given the conceptual and hypothesis-generating nature of this review, formal systematic review procedures (e.g., PRISMA framework) were not applied.

Study selection and prioritization were based on methodological quality and relevance to the central theme of perioperative immunometabolic modulation. Particular emphasis was placed on:

- Large randomized controlled trials evaluating oncologic outcomes;
- High-quality meta-analyses;
- Translational studies exploring perioperative immune and inflammatory mechanisms.

The objective was not to perform a quantitative synthesis, but to critically appraise existing evidence and identify conceptual and methodological gaps that may explain the discrepancy between biological plausibility and clinical trial findings.

### 1.2. The Perioperative Window as a Biological Turning Point

Surgical resection remains the cornerstone of treatment for most solid malignancies. Paradoxically, however, the perioperative period represents a phase of profound biological instability. Major surgery induces a coordinated neuroendocrine and inflammatory stress response characterized by activation of the hypothalamic–pituitary–adrenal axis, sympathetic nervous system stimulation, and release of catecholamines and glucocorticoids [7]. This systemic response, while adaptive in the context of tissue injury, exerts measurable effects on innate and adaptive immunity. Transient suppression of natural killer (NK) cell activity, alterations in T-cell subsets, and expansion of pro-inflammatory cytokine profiles have all been documented in the immediate postoperative period [8]. Such acute inflammatory perturbations may transiently reconfigure the tumor microenvironment at distant sites, potentially lowering the threshold for metastatic seeding during this vulnerable biological window.

Concurrently, tumor-related processes may be particularly sensitive to this altered host environment [9]. Circulating tumor cells, shed either spontaneously or during surgical manipulation, enter a systemic milieu marked by endothelial activation, platelet aggregation, and inflammatory signaling. Neutrophil extracellular trap (NET) formation, cytokine-driven vascular permeability, and stress-mediated β-adrenergic signaling may collectively facilitate tumor cell adhesion, extravasation, and early metastatic niche formation [10,11]. The perioperative period thus represents a transient but potentially critical window during which host–tumor interactions are dynamically reshaped [12].

Importantly, these immunologic and metabolic perturbations are not solely consequences of surgical trauma. Anesthetic and analgesic strategies contribute to the magnitude and duration of perioperative immune modulation. Volatile anesthetics, opioids, depth of anesthesia, and adequacy of analgesia may each influence inflammatory signaling pathways, stress hormone release, and cellular immune competence [13,14]. From this perspective, anesthesia is not biologically inert; rather, it becomes one of several determinants of the perioperative host response.

**Figure 1:**
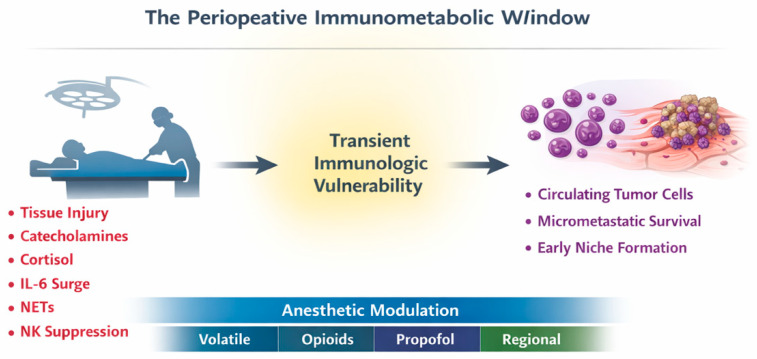
Conceptual model of the perioperative immunometabolic window. Illustration of the biological cascade triggered by surgical stress, including sympathetic activation, inflammatory cytokine release, neutrophil extracellular trap formation, and transient immune suppression, which together create a permissive environment for circulating tumor cell survival and early metastatic niche formation.

Within this framework, the concept of “immunometabolic reprogramming” gains relevance. Acute surgical stress alters glucose metabolism, mitochondrial function, and cytokine-mediated cellular energetics, potentially influencing immune cell function and tumor cell survival pathways [14,15]. Whether these transient shifts translate into durable oncologic consequences remains uncertain. However, the biological plausibility that perioperative stress and inflammation modulation could influence early micrometastatic dynamics warrants careful consideration [16]. The perioperative window should therefore be viewed not merely as a logistical interval surrounding surgery, but as a biologically active phase that may shape downstream oncologic trajectories [17,18]. The conceptual framework of this perioperative immunometabolic window is illustrated in Figure 1.

Importantly, most clinical trials evaluating anesthetic technique and oncologic outcomes have not incorporated perioperative immune or inflammatory phenotyping [4,5,19,20]. Systemic inflammatory indices such as the neutrophil-to-lymphocyte ratio (NLR) or the systemic immune–inflammation index (SII), as well as cytokine profiles including interleukin-6 and tumor necrosis factor-α, have been associated with oncologic prognosis across multiple malignancies [21,22]. Yet these parameters are rarely assessed longitudinally in the immediate perioperative period in the context of anesthetic intervention. The absence of biologically anchored endpoints may therefore represent a critical limitation of prior studies. Without defining the inflammatory and immunologic landscape in which anesthetic modulation occurs, it remains difficult to identify which patients—if any—might derive meaningful benefit from stress-attenuating strategies such as regional anesthesia.

### 1.3. Anesthetic Techniques as Biological Modifiers

Anesthetic management during oncologic surgery encompasses a spectrum of pharmacologic and regional strategies, each with potential implications for perioperative host–tumor interactions [23]. While traditionally viewed through the lens of hemodynamic stability, analgesia, and recovery profiles, anesthetic techniques also exert measurable effects on immune signaling, inflammatory cascades, and cellular stress pathways [24]. In the context of the biologically vulnerable perioperative window, these effects warrant closer examination.

Volatile anesthetics have been implicated in modulating hypoxia-inducible pathways, angiogenic signaling, and immune cell function in experimental models [16,25]. Activation of hypoxia-inducible factor-1α (HIF-1α) and downstream vascular endothelial growth factor (VEGF) expression has been described in certain in vitro and animal studies, suggesting a potential influence on tumor cell survival and angiogenesis [26,27,28]. However, translational consistency remains limited, and clinical evidence linking volatile agents to adverse oncologic outcomes is inconclusive [29]. The complexity of tumor biology and the multifactorial determinants of recurrence likely dilute any isolated pharmacologic effect [30].

Opioids, widely used for perioperative analgesia, have also been examined for their potential immunomodulatory properties [23,31]. Experimental data suggest that μ-opioid receptor activation may influence NK cell activity, T-cell function, and tumor cell signaling pathways [32,33]. Nevertheless, the clinical relevance of these findings remains debated, particularly in the era of multimodal analgesia and opioid-sparing strategies [34]. It is increasingly recognized that total opioid exposure, rather than the presence or absence of a specific agent, may be more relevant when considering immune modulation [35].

Intravenous anesthetic agents, particularly propofol, have been associated with anti-inflammatory properties and differential effects on immune cell activity compared with volatile agents in laboratory settings [28,36]. Yet, large randomized trials comparing propofol-based total intravenous anesthesia with volatile anesthesia have not consistently demonstrated survival advantages in major oncologic populations [37,38,39]. These findings reinforce the notion that isolated anesthetic components may exert subtle biological effects that are difficult to translate into uniform long-term clinical outcomes without accounting for tumor type, host biology, and perioperative inflammatory state [40].

Within this broader landscape, regional anesthesia occupies a distinctive conceptual position [41]. By attenuating afferent nociceptive signaling and blunting sympathetic activation, regional techniques may reduce the magnitude of the surgical stress response. Clinical and experimental studies suggest that regional anesthesia can be associated with reductions in perioperative cortisol levels of approximately 20–40%, decreased circulating catecholamine concentrations, and attenuation of interleukin-6 (IL-6) peaks by 30–50%, depending on the surgical model and technique used [42]. These mechanisms provide a biologically coherent rationale for potential modulation of perioperative immune function [43]. However, such plausibility should not be conflated with proven oncologic benefit [44]. Rather than functioning as an independent anti-tumor therapy, regional anesthesia may act as one component within a multimodal strategy aimed at stabilizing the perioperative immunometabolic environment [45].

### 1.4. Why Have Large Clinical Trials Failed to Demonstrate Survival Benefit?

Several large randomized controlled trials have sought to determine whether anesthetic technique influences long-term oncologic outcomes [42,46]. For example, in the randomized trial by Sessler et al. [2], breast cancer recurrence occurred in approximately 10% of patients in both groups at 3 years, with no statistically significant difference between regional and general anesthesia. Similarly, the study by Badwe et al. [18] reported disease-free survival rates exceeding 90% at 5 years in both the intervention and control groups, with no detectable effect of local anesthetic infiltration. These findings highlight that, despite adequate statistical power, absolute differences in outcomes were small and potentially obscured by biological heterogeneity. Representative clinical evidence is summarized in Table 1.

Despite compelling biological hypotheses and early observational signals, these trials have generally failed to demonstrate consistent reductions in cancer recurrence or improvements in overall survival associated with regional anesthesia or specific anesthetic regimens [13,18,34,35,47,48]. At face value, these findings might suggest that anesthetic modulation is clinically irrelevant. However, a closer examination suggests that the negative results may reflect methodological and conceptual limitations rather than a definitive lack of biological neutrality [49].

First, oncologic endpoints, such as disease-free or overall survival, are temporally and biologically distinct from perioperative interventions [25]. Cancer recurrence is influenced by tumor genomics, stage, margin status, adjuvant therapy, host immune competence, and comorbidities—variables that unfold over years. In contrast, anesthetic exposure occurs within hours [46]. Expecting a brief perioperative intervention to uniformly alter long-term survival across heterogeneous malignancies may overextend mechanistic plausibility. The absence of a detectable effect at five years does not necessarily exclude meaningful biological modulation within the immediate postoperative period [1,4,16,50].

Second, most trials have treated malignancies as homogeneous entities, enrolling broad patient populations without biological stratification [49,51]. Yet tumor subtypes differ profoundly in their immunogenicity, metastatic patterns, and responsiveness to inflammatory cues [52]. Likewise, host inflammatory phenotypes vary substantially [53,54]. Without identifying patients characterized by heightened perioperative inflammatory activation or immunologic vulnerability, any potential benefit of stress-attenuating strategies may be diluted within a heterogeneous cohort [55].

Third, perioperative care has evolved. Enhanced recovery pathways, multimodal analgesia, reduced opioid exposure, and refined surgical techniques have collectively attenuated the magnitude of surgical stress [56]. In such optimized settings, the incremental contribution of regional anesthesia may be smaller than in earlier eras. Large trials conducted within standardized ERAS frameworks may therefore compare two relatively stress-mitigated strategies, reducing the likelihood of observing divergence in long-term outcomes [57,58].

Finally, most studies have focused exclusively on distant oncologic endpoints without incorporating mechanistic intermediate outcomes. The absence of perioperative immune profiling, inflammatory markers, or translational correlates limits the ability to interpret negative findings [42,51]. Without confirming whether an anesthetic intervention meaningfully altered the perioperative immunologic landscape, it becomes difficult to determine whether a true biological effect was absent or simply unmeasured [26].

**Figure 2:**
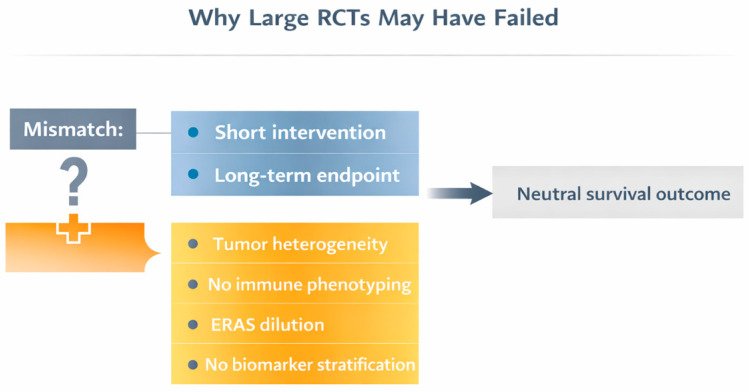
Methodological mismatch between anesthetic intervention and long-term oncologic endpoints. Schematic representation of the conceptual limitations of prior randomized trials evaluating anesthetic technique and cancer recurrence. The figure highlights the temporal disconnect between short perioperative anesthetic exposure and distant survival endpoints, the lack of inflammatory or immune phenotyping, and the dilution of potential effects within heterogeneous tumor populations.

Taken together, these considerations suggest that the failure to demonstrate a survival benefit may not invalidate the biological rationale for perioperative modulation [59]. Rather, it underscores the need for a refined investigative framework—one that aligns mechanistic hypotheses with biologically proximal endpoints and stratifies patients according to tumor and host inflammatory characteristics [25]. The principal methodological and conceptual mismatches underlying prior neutral trials are summarized in Figure 2.

### 1.5. Toward a New Investigative Framework

If anesthetic modulation of oncologic outcomes is to be meaningfully evaluated, future investigations must more closely align mechanistic hypotheses with study design. Rather than framing the question solely in terms of long-term recurrence, research should focus on biologically proximal endpoints within the perioperative window [60,61,62]. This shift does not diminish the importance of survival outcomes but recognizes that transient immunologic and inflammatory perturbations are the most immediate and measurable effects of anesthetic interventions [43,63,64].

A biologically informed framework would incorporate perioperative immune and inflammatory phenotyping as integral components of trial design [55]. Serial assessment of systemic inflammatory indices, cytokine dynamics, stress hormone profiles, and markers of innate immune function could help determine whether specific anesthetic strategies meaningfully attenuate the perioperative stress response [43]. Such intermediate endpoints would provide mechanistic clarity and enable identification of patient subgroups characterized by heightened inflammatory activation or immunologic vulnerability [65]. Key distinctions between traditional survival-oriented trial designs and biologically informed perioperative models are outlined in Table 2.

In this context, anesthetic technique becomes a modulator of host response rather than a presumed determinant of long-term survival [16,26].

Equally important is the recognition of tumor heterogeneity. Future studies should consider stratifying by tumor immunogenicity, stage, molecular subtype, and anticipated adjuvant therapy [66]. It is plausible that any benefit of perioperative stress attenuation would be most relevant in biologically aggressive tumors with high metastatic potential or in patients with pre-existing inflammatory dysregulation [67]. A precision-based approach—integrating tumor biology with host inflammatory phenotype—may therefore be necessary to detect clinically meaningful signals [68].

Moreover, anesthetic interventions should be conceptualized as components of a broader perioperative immunomodulatory strategy [63,69]. Regional anesthesia, opioid-sparing analgesia, hemodynamic stability, glycemic control, and minimization of excessive inflammatory activation are interrelated elements of a unified physiological response [70]. Evaluating these factors in isolation risks oversimplifying a complex biological system. A systems-oriented model that incorporates multimodal modulation of perioperative stress and metabolism may better reflect real-world clinical practice and biological reality [71,72].

Ultimately, the goal is not to reposition regional anesthesia as an anti-cancer therapy, but to situate anesthetic management within the broader landscape of perioperative oncology [71]. By acknowledging the transient yet potentially consequential nature of perioperative immune perturbations, future research can move beyond binary conclusions toward a more nuanced understanding of how anesthetic care interfaces with tumor biology [25].

## 2. Limitations

This review has several limitations. First, as a narrative review, it does not follow a formal systematic methodology and is therefore subject to potential selection bias. Although a structured search strategy was applied, study inclusion was not based on predefined systematic criteria, which may have influenced the representation of the available evidence.

Second, the included studies are heterogeneous with respect to tumor types, anesthetic techniques, perioperative management strategies, and outcome definitions. This variability limits the ability to draw uniform conclusions and reflects the complexity of the field.

Third, much of the available evidence derives from observational studies and translational or experimental models, with a relative paucity of randomized trials that incorporate biologically integrated endpoints. As a result, causal relationships between anesthetic modulation and oncologic outcomes remain difficult to establish.

Finally, a fundamental limitation is the challenge of translating short-term perioperative immunologic and inflammatory changes into long-term oncologic outcomes, such as recurrence or survival. This translational gap represents not only a limitation of the existing evidence but also a central theme of this review, highlighting the need for future studies that align mechanistic insights with clinically meaningful endpoints.

## 3. Conclusions

### Future Directions and Clinical Implications

The expanding field of perioperative medicine increasingly recognizes surgery not merely as a technical intervention, but as a systemic biological event. In oncologic care, this perspective invites reconsidering the perioperative period as a potentially modifiable interface between tumor biology and host physiology [73]. As immunotherapy, targeted treatments, and personalized oncology continue to evolve, the perioperative phase may emerge as an underexplored determinant of therapeutic synergy or vulnerability [16].

Future research should therefore aim to integrate anesthetic strategy into broader perioperative immunologic optimization [55]. Trials incorporating immune profiling, inflammatory signatures, and translational biomarkers may clarify whether modulation of surgical stress meaningfully influences early tumor–host dynamics. Importantly, such investigations should be hypothesis-driven and biologically stratified, rather than broadly applied across heterogeneous malignancies [63]. Precision in patient selection and endpoint definition will be essential to avoid repeating the conceptual limitations of prior large-scale trials.

In parallel, clinical practice may benefit from reframing anesthetic management in oncologic surgery as part of a multimodal physiological stabilization strategy [19]. Attenuation of excessive stress responses, minimization of opioid exposure, maintenance of metabolic equilibrium, and preservation of immune competence represent interdependent goals that extend beyond immediate postoperative recovery [43]. Whether these measures ultimately translate into measurable oncologic benefit remains to be established; however, their biological plausibility and alignment with principles of enhanced recovery provide a coherent rationale for continued investigation [69].

Importantly, future studies should report not only statistical significance but also the magnitude of perioperative immunologic changes and their potential clinical relevance.

Regional anesthesia, within this context, should neither be dismissed as ineffective nor promoted as an anti-cancer intervention [74]. Instead, it may be most appropriately viewed as one element within a systems-based approach to perioperative care—an approach that acknowledges the transient yet biologically active nature of the surgical interval [75]. Advancing this field will require interdisciplinary collaboration between anesthesiologists, oncologists, immunologists, and translational scientists, moving the discussion from binary outcomes toward mechanistic clarity.

The perioperative window is brief, but biologically intense. Understanding how anesthetic management interacts with this window may not redefine oncology, but it may refine the anesthesiologist’s role within it.

## Figures and Tables

**Table 1:** Key clinical evidence on anesthetic techniques and oncologic outcomes.

Study (Year)	Cancer Type	Intervention	Control	Primary Endpoint	Main Result	Key Quantitative Findings	Key Methodological Considerations
Sessler et al. [2], 2019 The Lancet	Breast cancer	Paravertebral block + propofol	Sevoflurane + opioids	Cancer recurrence	No difference	~10% recurrence in both groups	Heterogeneous tumor biology; no perioperative immune profiling; long-term endpoint disconnected from perioperative window
Badwe et al. [18], 2023 Journal of Clinical Oncology	Early breast cancer	Peritumoral local anesthetic infiltration	Placebo infiltration	Disease-free survival	No difference	>90% DFS at 5 years	Single local intervention; no systemic inflammatory assessment
Han et al. [47], 2024 Regional Anesthesia & Pain Medicine	Bladder cancer (NMIBC)	Regional anesthesia	General anesthesia	2-year recurrence	No significant difference	Similar recurrence rates between groups (~20–30% overall cohort)	Short follow-up; heterogeneous risk categories; no immune stratification
Li et al. [42], 2023 World Journal of Surgical Oncology	Mixed solid tumors (meta-analysis of RCTs)	Regional anesthesia	General anesthesia	Long-term survival	No survival benefit	HR ~1.0 (no effect)	Aggregated heterogeneous malignancies; lack of mechanistic endpoints

**Table 2:** Conceptual differences between mechanistic and survival-oriented trial designs.

Traditional Design	Biologically Informed Design
Endpoint: 5-year recurrence	Endpoint: perioperative immune modulation
No biomarker stratification	Inflammatory phenotyping
Heterogeneous tumors	Molecular/tumor subtype stratification
Binary comparison	Systems-based multimodal model

## Data Availability

No new data were generated or analyzed in support of this review. Data sharing is not applicable to this article.
